# CD4^+^ and CD8^+^ T Cells Can Act Separately in Tumour Rejection after Immunization with Murine Pneumotropic Virus Chimeric Her2/*neu* Virus-Like Particles

**DOI:** 10.1371/journal.pone.0011580

**Published:** 2010-07-19

**Authors:** Kalle Andreasson, Mathilda Eriksson, Karin Tegerstedt, Torbjörn Ramqvist, Tina Dalianis

**Affiliations:** Department of Oncology-Pathology, Karolinska Institutet, Stockholm, Sweden; University of California Los Angeles, United States of America

## Abstract

**Background:**

Immunization with murine pneumotropic virus virus-like particles carrying Her2/*neu* (Her2MPtVLPs) prevents tumour outgrowth in mice when given prophylactically, and therapeutically if combined with the adjuvant CpG. We investigated which components of the immune system are involved in tumour rejection, and whether long-term immunological memory can be obtained.

**Methodology and Results:**

During the effector phase in BALB/c mice, only depletion of CD4^+^ and CD8^+^ in combination, with or without NK cells, completely abrogated tumour protection. Depletion of single CD4^+^, CD8^+^ or NK cell populations only had minor effects. During the immunization/induction phase, combined depletion of CD4^+^ and CD8^+^ cells abolished protection, while depletion of each individual subset had no or negligible effect. When tumour rejection was studied in knock-out mice with a C57Bl/6 background, protection was lost in CD4^−/−^CD8^−/−^ and CD4^−/−^, but not in CD8^−/−^ mice. In contrast, when normal C57Bl/6 mice were depleted of different cell types, protection was lost irrespective of whether only CD4^+^, only CD8^+^, or CD4^+^ and CD8^+^ cells in combination were eradicated. No anti-Her2/*neu* antibodies were detected but a Her2/*neu*-specific IFNγ response was seen. Studies of long-term memory showed that BALB/c mice could be protected against tumour development when immunized together with CpG as long as ten weeks before challenge.

**Conclusion:**

Her2MPtVLP immunization is efficient in stimulating several compartments of the immune system, and induces an efficient immune response including long-term memory. In addition, when depleting mice of isolated cellular compartments, tumour protection is not as efficiently abolished as when depleting several immune compartments together.

## Introduction

Different forms of immunotherapeutic strategies against cancer are constantly being investigated as complements to conventional cancer treatments. The proto-oncogene Her2/*neu* [1] is an attractive tumour antigen for immunotherapy against breast cancer since it is overexpressed in some 20% of all breast cancers and in addition, the transformed phenotype is dependent on its expression. Moreover, immune responses to Her2/*neu* are frequently found in patients with Her2/*neu* positive breast cancer, proving that tolerance to this tumour antigen can be broken in humans [2], [3].

Capsid proteins of some viruses can spontaneously self-assemble into viral capsids known as virus-like particles (VLPs) [4]–[6], which can be used as vaccines and vectors for gene and immune therapy. The best known are the VLP vaccines against human papillomavirus (HPV) types 6, 11, 16 and 18 [7]. Additionally, vaccination with chimeric VLPs (cVLPs) carrying various tumour antigens has been shown to prevent tumour outgrowth in different mouse models [8]–[11].

We have previously used cVLPs based on murine polyomavirus and murine pneumotropic virus as vaccines against Her2/*neu* expressing tumours. These cVLPs carry a fusion protein between the C-terminal part of the VP2 protein and the extracellular and transmembrane domains of human Her2/*neu* (Her2MPyVLPs and Her2MPtVLPs, respectively). We have shown that a single prophylactic vaccination with Her2MPyVLPs or Her2MPtVLPs protected >90% of mice against outgrowth of a tumour cell line expressing the homologous human Her2/*neu* protein (D2F2/E2) [8], [11]. In addition, therapeutic immunization with Her2MPtVLPs together with the adjuvant CpG as late as six days after challenge resulted in 80% tumour-free mice [11].

The immune response was demonstrated to be Her2/*neu*-specific since Her2MPyVLP immunization did not induce rejection of the parental D2F2 cell line (lacking the transfected Her2/*neu* gene) [8]. Moreover, splenocytes from Her2MPyVLP immunized mice secreted IFNγ when incubated together with D2F2/E2, or CD8^+^ Her2/*neu* immunodominant epitopes, but not when incubated with D2F2 cells or control peptide epitopes as shown by ELISPOT [8], [11]. However, an antibody response to Her2/*neu* was never demonstrated in this system [8], [11], [12].

Together the findings *in vitro* indicated that tumour rejection using Her2MPyVLPs and Her2MPtVLPs was primarily mediated by CD8^+^ cytotoxic T cells, but whether other immune cells were also involved was never pursued. Further information regarding these immune mechanisms *in vivo*, when using Her2/*neu*-cVLPs, including studies on long-term memory would be of great value to further improve the efficacy of vaccination.

In this study we have elucidated which components of the immune system that are important for tumour protection, both in the induction and the effector phases, after vaccination with cVLPs from murine pneumotropic virus. The effects of Her2MPtVLP vaccination were followed in BALB/c and C57Bl/6 mice. Depletion of CD4^+^, CD8^+^ or both CD4^+^ and CD8^+^ T cells, as well as NK cells, was performed to find out which of these cell types are of importance for the obtained immunity. In parallel, C57Bl/6 knock-out mice lacking CD4^+^, CD8^+^ or both CD4^+^ and CD8^+^ T cells were used. We have also investigated whether long-term immunological memory can be induced by Her2MPtVLPs.

## Materials and Methods

### Ethics statement

CD4^−/−^, CD8^−/−^ and CD4^−/−^CD8^−/−^ C57Bl/6 (H-2^b^) mice were with C57Bl/6 and BALB/c (H-2^d^), bred and maintained at MTC Karolinska Institutet and the project conducted according to Ethical permission N331/08 from Stockholms Norra Djurforsoksetiska Namnd, Sweden.

### Antibodies and reagents

Anti-CD4 (clone GK1.5) and anti-CD8 (clone TIB105) monoclonal antibodies (mAbs) were purchased from Mabtech (Nacka, Sweden). Anti-asialo GM1 polyclonal antibody was obtained from Wako Chemicals (Neuss, Germany). Anti-CD4-FITC, anti-CD8-FITC, anti-CD3-FITC and anti-CD49b-PE (clone DX5) as well as anti-Her2/*neu*-PE and its isotype control were obtained from BD Biosciences Pharmingen (San Diego, CA, USA). CpG 1826 was obtained from CyberGene (Stockholm, Sweden).

### Cell lines

D2F2/E2 cells (H-2^d^) expressing the human Her2/*neu* protein [13] were cultured as described [12]. EL4 cells (H-2^b^) [14] were transfected with the pcDNA3/HER-2 plasmid (harbouring full-length human Her2/*neu* from pVAX/E2A [15] and the neomycin resistance gene) using Lipofectamine 2000 reagent (Invitrogen, Carlsbad, CA, USA), according to the manufacturer's instructions, to obtain a Her2/*neu* positive H-2^b^ cell line. Cells were cloned and analysed for surface expression of Her2/*neu* by flow cytometry using the anti-Her2/*neu*-PE antibody. The clone (EL4-Her2) with the strongest Her2/*neu* expression was expanded and passaged once *in vivo* to improve its quality and then maintained in Dulbecco's modified Eagle's medium supplemented with 10% FCS, penicillin/streptomycin and 800 µg/ml G418 antibiotic solution.

### Construction of VLPs

The generation and purification of Her2MPtVLPs and the control MPtVLPs have been described previously [11], [16].

### Immunization and tumour cell challenge

Groups of 6–10 female BALB/c or C57Bl/6 mice were given a single subcutaneous immunization with 50 µg Her2MPtVLPs and challenged 14 days later subcutaneously in the flank with 5×10^4^ D2F2/E2 cells or 1–2×10^3^ EL4-Her2 cells. Tumour size was measured twice a week and mice carrying a tumour >1 mm^3^ were considered as tumour-bearing. Mice were sacrificed when the largest tumour diameter reached 10 mm. For long-term memory experiments, groups of 10 female BALB/c mice were immunized with 50 µg Her2MPtVLPs alone, or together with 50 µg CpG, 10 or 6 weeks before challenge. As a positive control, immunization with 50 µg Her2MPtVLPs was carried out 14 days prior to tumour challenge.

### *In vivo* depletion of immune cell subsets during the immunization or effector phases

For depletion of CD4^+^ and CD8^+^ cells in the immunization phase, mice were given intraperitoneal (i.p.) injections of 200 µg anti-CD4 and/or anti-CD8 mAbs four days and one day before, and two days after immunization. Depletion was analysed by flow cytometry four days after one single injection by staining splenocytes with FITC-conjugated anti-CD4 and anti-CD8 antibodies. The level of depletion was >97% for both T-cell subsets.

For depletion of CD4^+^ and/or CD8^+^ cells (and in BALB/c also of NK cells) in the effector phase, mice were injected i.p. with 200 µg anti-CD4 mAb, 200 µg anti-CD8 mAb and/or 20 µl anti-asialo GM1 antibody. The first depletion was performed two days before tumour cell challenge, and then every 4–5 days until day 50 after challenge. Depletion of both T-cell subsets was >97% when tested four days after one single injection and 70–80% at the day of termination of the experiment. NK cells decreased from 8–10% of splenocytes to 2–3% as analysed by flow cytometry using anti-CD49b (clone DX5) and anti-CD3 mAbs (NK cells being CD49b^+^CD3^−^). This level of depletion is in line with other reports [17]–[20].

### ELISPOT assay and measurement of Her2/*neu* antibodies

ELISPOT as well as measurement of Her2/*neu* antibodies were performed as described previously [11].

### Statistical analyses

Student's t-test was used to analyse differences in ELISPOT data. Differences in tumour takes were analysed using Fisher's exact two-tailed test or, when indicated, the Peto-Peto-Wilcoxon test.

## Results

### Depletion of both CD4^+^ and CD8^+^ T cells is necessary to abolish the induction of an immune response in BALB/c mice

**Figure 1:**
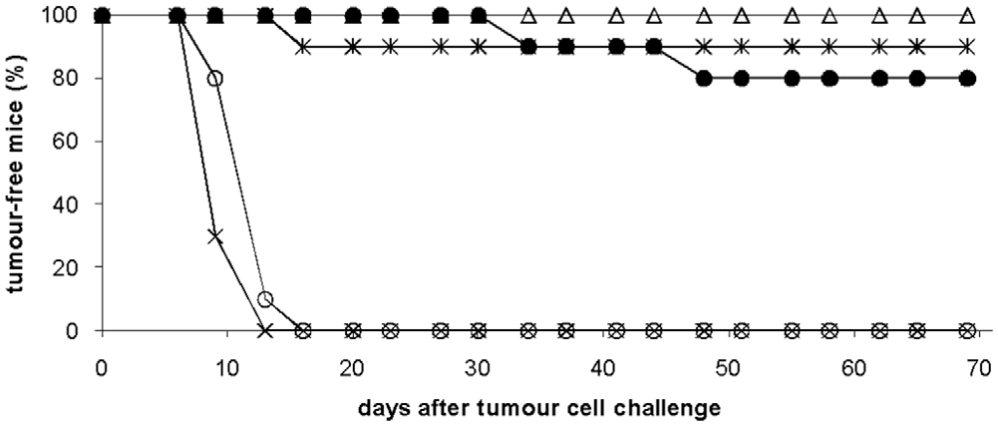
Tumour rejection following depletion of immune cells in the induction phase in BALB/c mice. Mice immunized with Her2MPtVLPs were depleted of CD4^+^ cells (•), CD8^+^ cells (*), or both cell types (O), 4 days and 1 day before immunization, and 2 days after immunization, respectively. Unimmunized mice (x) and non-depleted Her2MPtVLP immunized mice (Δ) were included as negative and positive controls respectively. Mice were challenged with 5×10^4^ D2F2/E2 cells 2 weeks after immunization.

BALB/c mice were depleted of CD4^+^ and/or CD8^+^ cells in the induction phase using i.p. injections of mAbs days −4, −1 and +2 in relation to Her2MPtVLP immunization. Mice were inoculated with 5×10^4^ D2F2/E2 tumour cells 14 days after immunization and followed for tumour outgrowth (Figure 1). Her2MPtVLP immunized non-depleted mice stayed tumour-free throughout the 69-day observation period, while all unimmunized control mice developed tumours within 13 days. A considerable immunization effect was still observed after CD4^+^ or CD8^+^ depletion, since 80% and 90% of the mice respectively stayed tumour-free during the whole observation period (p<0.001 compared to unimmunized mice in both cases). However, the protective effect was abolished when both CD4^+^ and CD8^+^ cells were depleted, and all mice developed tumours in a timeframe similar to that in unimmunized animals.

**Figure 2:**
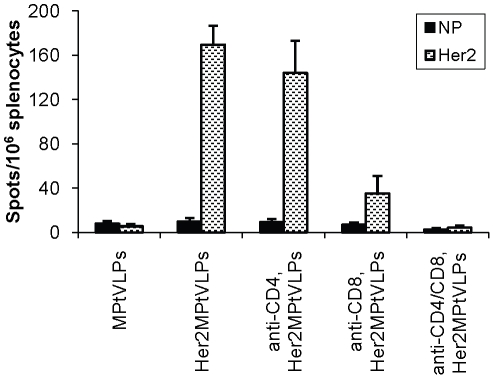
IFNγ responses following depletion of immune cells in BALB/c mice. Mice were depleted of CD4^+^ and/or CD8^+^ cells 5 and 2 days before immunisation with Her2MPtVLPs. The IFNγ response was measured 7 days later by stimulating splenocytes with the immunodominant CD8^+^ peptide Her2_63–71_ or the control peptide NP_118–126_. Average values of triplicates from six animals are shown. Background values (stimulation in the absence of peptide) have been subtracted. Error bars represent S.E.

The *in vivo* data above were complemented with the monitoring of IFNγ responses by ELISPOT. Mice were depleted of CD4^+^ and/or CD8^+^ cells 5 days and 2 days before immunization, and thereafter immunized with Her2MPtVLPs. ELISPOT analyses were performed 7 days later. Depletion of CD4^+^ cells did not affect the induced IFNγ response as compared to non-depleted mice (p = 0.68) (Figure 2). As expected, depletion of CD8^+^ cells, or depletion of CD4^+^ in combination with CD8^+^ cells, almost completely abolished the IFNγ response (p<0.01 in both cases) (Figure 2).

### Depletion of both CD4^+^ and CD8^+^ T cells in the effector phase is necessary to abolish tumour rejection after Her2MPtVLP vaccination in BALB/c mice

BALB/c mice were immunized with Her2MPtVLPs and challenged with 5×10^4^ D2F2/E2 tumour cells 14 days later. CD4^+^ cells, CD8^+^ cells or NK cells were depleted separately or together by i.p. injections of monoclonal antibodies (for CD4^+^ and CD8^+^ cells), or anti-asialo GM1 antibodies (for NK cells), every 4–5 days starting two days before challenge. The experiment was performed twice with similar results, and the results are summarised in Table 1. Non-depleted Her2MPtVLP immunized mice remained tumour-free, while all but one unimmunized control mouse developed tumours. Depletion of CD4^+^ and CD8^+^ cells in combination, with or without depletion of NK cells, almost completely abolished the anti-tumour effect. Single depletion of CD4^+^, CD8^+^ or NK cells still resulted in a statistically significant anti-tumour response compared to unimmunized mice. However, depletion of either CD4^+^ cells or NK cells resulted in reduced protection compared to non-depleted immunized mice (Table 1).

**Table 1:** Tumour takes after depletion of CD4^+^ cells, CD8^+^ cells and/or NK cells in the effector phase in BALB/c and C57Bl/6 mice, respectively, as well as tumour takes in C57Bl/6 knock-out mice.

Mouse strain	Immunogen	Cells depleted	Takes		Total takes (%)	p-values^a^	p-values^b^
			Exp.^c^ 1	Exp. 2			
**BALB/c**	**Her2MPtVLPs**	**-**	0/10	0/10	0/20 (0)	<0.001	
	**Her2MPtVLPs**	**CD4^+^**	3/10	3/9	6/19 (32)	<0.001	<0.01
	**Her2MPtVLPs**	**CD8^+^**	2/10	0/10	2/20 (10)	<0.001	ns^d^
	**Her2MPtVLPs**	**CD4^+^/CD8^+^**	8/10	7/10	15/20 (75)	ns	<0.001
	**Her2MPtVLPs**	**CD4^+^/CD8^+^/NK**	9/10	6/10	15/20 (75)	ns	<0.001
	**Her2MPtVLPs**	**NK**	4/10	8/10	12/20 (60)	<0.05	<0.001
	**-**	**-**	10/10	9/10	19/20 (95)		<0.001
**B6** e **wild-type**	**-**	**-**	5/7	8/10	13/17 (76)		
	**Her2MPtVLPs**	**-**	2/7	3/10	5/17 (29)	<0.05	
**B6 CD4^+^ KO** f	**-**	**-**	4/7	5/6	9/13 (69)		
	**Her2MPtVLPs**	**-**	4/7	4/6	8/13 (62)	ns	
**B6 CD8^+^ KO**	**-**	**-**	5/7	7/10	12/17 (71)		
	**Her2MPtVLPs**	**-**	0/7	3/10	3/17 (18)	<0.01	
**B6 CD4^+^/CD8^+^ KO**	**-**	**-**	6/7	9/9	15/16 (94)		
	**Her2MPtVLPs**	**-**	6/7	8/9	14/16 (88)	ns	
**B6 wild-type**	**Her2MPtVLPs**	**CD4^+^**	6/7		6/7 (86)	ns	<0.05
	**Her2MPtVLPs**	**CD8^+^**	5/7		5/7 (71)	ns	ns
	**Her2MPtVLPs**	**CD4^+^/CD8^+^**	5/7		5/7 (71)	ns	ns

Mice were immunized with Her2MPtVLPs and 2 weeks later challenged with 5×10^4^ D2F2/E2 cells (BALB/c) or 1–2×10^3^ EL4-Her2 cells (C57Bl/6). P-values were calculated using Fisher's exact two-tailed test for BALB/c mice depleted of different cells as well as for C57Bl/6 knock-out mice while Peto-Peto-Wilcoxon was used for C57Bl/6 mice depleted of various cell types.

a compared to unimmunized mice.

b compared to non-depleted immunized mice.

c experiment.

d not significant.

e C57Bl/6 mice.

f knock-out.

### Tumour rejection by Her2MPtVLPs is induced in CD8^−/−^ single, but not in CD4^−/−^ single or CD4^−/−^CD8^−/−^ double knock-out C57Bl/6 mice

**Figure 3:**
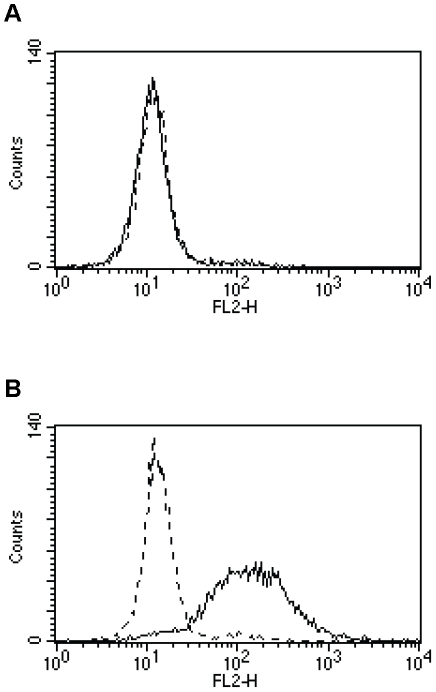
Surface expression of Her2/*neu* on EL4-Her2 cells. Surface expression of human Her2/*neu* on EL4 (A), and EL4-Her2 cells (B), analysed by flow cytometry using the anti-Her2/*neu*-PE antibody (solid line) or relevant isotype control (dashed line).

As a complement to the depletion of different subsets of immune cells in normal BALB/c mice, tumour rejection experiments were also performed in CD4^−/−^ single, CD8^−/−^ single, or CD4^−/−^CD8^−/−^ double knock-out C57Bl/6 mice. EL4-Her2, derived from EL4 cells (H-2^b^) transfected with a plasmid expressing human Her2/*neu*, were used for tumour challenge. Surface expression of Her2/*neu* was re-confirmed by flow cytometry prior to tumour challenge (Figures 3a and b). To ensure a positive immune response, normal C57Bl/6 mice, in parallel to C57Bl/6 knock-out mice, were immunized subcutaneously with Her2MPtVLPs and challenged with EL4-Her2 cells two weeks later. The results from two independent experiments are summarized in Table 1. Her2MPtVLP immunized normal and CD8^−/−^ single knock-out C57Bl/6 mice were protected against tumour outgrowth (p<0.05 and p<0.01, respectively), indicating that these groups of mice could mount an immune response after Her2MPtVLP immunization, while most unimmunized mice developed tumours (Table 1). In CD4^−/−^ single and CD4^−/−^CD8^−/−^ double knock-out C57Bl/6 mice, there was no significant difference in tumour takes between Her2MPtVLP immunized and unimmunized mice (Table 1).

### Depletion of either CD4^+^ or CD8^+^ cells in the effector phase completely abrogates tumour rejection in C57Bl/6 mice

Since the results from the depletion of immune cells in BALB/c mice were not completely in agreement with the results from the C57Bl/6 knock-out mice, we performed a depletion experiment in C57Bl/6 mice. C57Bl/6 mice were immunized with Her2MPtVLPs (50 µg) and challenged with 2×10^3^ EL4-Her2 cells 14 days later. CD4^+^ and CD8^+^ cells were depleted separately or together using i.p. injections of anti-CD4 and anti-CD8 mAbs starting two days before tumour cell challenge, and thereafter every 4–5 days. EL4-Her2 tumour outgrowth in the different groups is shown in Table 1. In this system, where the degree of immunity was less pronounced, depletion of CD4^+^ cells or CD8^+^ cells, or CD4^+^ and CD8^+^ cells in combination, abolished the Her2MPtVLP immunization effect completely (Table 1). However, only depletion of CD4^+^ cells resulted in a statistically significant difference compared to non-depleted Her2MPtVLP immunized mice (p<0.05, Peto-Peto-Wilcoxon test).

### Her2MPtVLP immunization induces long-term T cell memory responses

**Figure 4:**
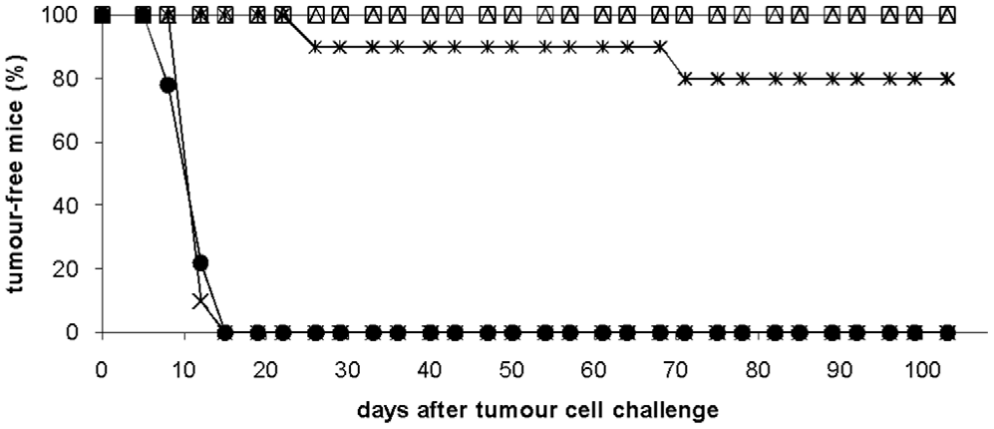
Induction of long-term tumour rejection responses. BALB/c mice were immunized with Her2MPtVLPs alone (*) or Her2MPtVLPs in combination with CpG (Δ) and challenged 6 weeks later with 5×10^4^ D2F2/E2 cells. Unimmunized mice (x) and mice immunized with MPtVLPs and CpG (•) were included as negative controls, while mice immunized with Her2MPtVLPs 2 weeks prior to challenge were used as positive controls (□).

**Figure 5:**
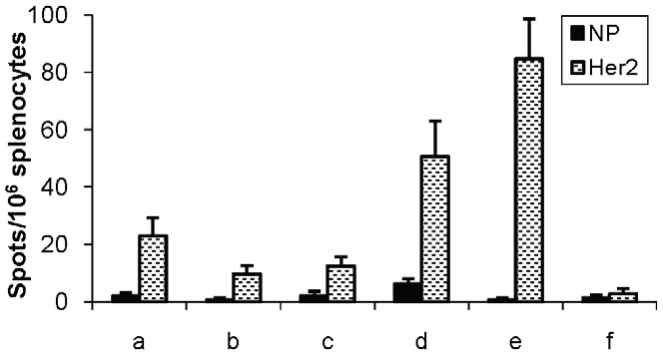
Long-term IFNγ responses after Her2MPtVLP immunization. BALB/c mice were immunized with Her2MPtVLPs with CpG either 10 weeks (a), 6 weeks (b) or 1 week (d) before ELISPOT analysis, or with Her2MPtVLPs without CpG 6 weeks (c) or 1 week (e) before ELISPOT. Unimmunized mice were included as negative controls (f). Splenocytes were stimulated with the immunodominant CD8^+^ peptide Her2_63–71_ or the control peptide NP_118–126_. Average values of triplicates from 4 animals are shown. Background values (stimulation in the absence of peptide) have been subtracted. Error bars represent S.E.

As demonstrated above and reported previously [8], [11], [12], inhibition of tumour outgrowth is regularly 95–100% when BALB/c mice are immunized with Her2/*neu*-cVLPs two weeks prior to challenge. Due to this successful anti-tumour response, we examined whether long-term immunological memory could be obtained. For this purpose, BALB/c mice were immunized with Her2MPtVLPs with or without CpG either six or ten weeks before challenge. Two experiments were performed and the results from the first experiment are shown in Figure 4 and the results from both experiments are summarised in Table 2. All but one unimmunized mouse developed tumours. Combining Her2MPtVLPs with CpG immunization six weeks before challenge resulted in the same level of protection as immunization two weeks prior to challenge (p<0.001 compared to unimmunized mice). Immunization with Her2MPtVLPs and CpG as long as ten weeks before challenge still resulted in significant protection compared to unimmunized mice (60% protection, p<0.01) (Table 2). Finally, when tumour cells were given six weeks after immunization with Her2MPtVLPs without CpG, protection was 80% (p<0.001) (Table 2). In accordance with these results, an IFNγ response specific for the immunodominant CD8^+^ peptide Her2_63–71_ could be demonstrated by ELISPOT up to ten weeks after immunization with Her2MPtVLPs and CpG. However, the response was weak at both six and ten weeks (Figure 5).

**Table 2:** Tumour takes after immunization of BALB/c mice 6 and 10 weeks before challenge with 5×104 D2F2/E2 cells.

Immunogen	Takes		Total takes (%)	p-values^a^
	Exp.^b^ 1	Exp. 2		
**Her2MPtVLPs+CpG, −10 weeks**	nd^c^	4/10	4/10 (40)	<0.01
**Her2MPtVLPs+CpG, −6 weeks**	0/10	1/10	1/20 (5)	<0.001
**Her2MPtVLPs, −6 weeks**	2/10	nd	2/10 (20)	<0.001
**Her2MPtVLPs+CpG, −2 weeks**	0/10	1/10	1/20 (5)	<0.001
**MPtVLPs+CpG, −2 weeks**	10/10	nd	10/10 (100)	ns^d^
**Unimmunized**	10/10	9/10	19/20 (95)	ref

a p-values calculated using Fisher's exact two-tailed test, compared to unimmunized mice.

b experiment.

c not done.

d not significant.

## Discussion

In this study, the role of CD8^+^, CD4^+^ and NK cells in tumour rejection was examined after Her2MPtVLP immunization. Previous reports strongly indicated that CD8^+^ T cells play a role, but the effect of other cell types was never followed [8], [11]. In addition, different ways to obtain long-term memory were explored.

In BALB/c mice immunized with Her2MPtVLP, depletion of both, but not only either CD4^+^ or CD8^+^ cells was required to eliminate inhibition of tumour growth, irrespective of whether the anti-CD4 and/or anti-CD8 mAbs were delivered before immunization, or before tumour challenge. Depletion of NK cells was only performed in the effector phase and did not add any effect to anti-CD4 and anti-CD8 antibody treatments, but did impair immunity when depleted alone. Tumour rejection experiments were also done in Her2MPtVLP immunized normal and CD4^−/−^ and CD8^−/−^ single, as well as CD4^−/−^CD8^−/−^ double knock-out C57Bl/6 mice. Similar to BALB/c mice, protection against tumour outgrowth was obtained in both normal and CD8^−/−^ mice, but not in CD4^−/−^CD8^−/−^ mice. However, in contrast to BALB/c mice, protection was lost in CD4^−/−^ mice, and after depletion of either CD4^+^ and/or CD8^+^ cells in normal C57Bl/6 mice.

The fact that tumour rejection was obtained after depletion of CD8^+^ cells in BALB/c mice was unexpected in comparison to our previous results [8], [11]. This could have been due to an antibody response. However, anti-Her2/*neu* antibodies have never been found using these vaccines [8], [11], [12]. In addition, the mice were still protected after CD4^+^ cell depletion at the time of immunization, suggesting that if antibodies were raised, this occurred independent of CD4^+^ cells. Moreover, tumour protection was lost after combined depletion of CD4^+^ and CD8^+^ cells, which should not have had a more profound effect on antibody titres than depletion of CD4^+^ cells alone. It is also unlikely that tumour inhibition after depletion was due to low numbers of remaining CD4^+^ or CD8^+^ specific T-cells, since depletion was >97% initially for both subsets, and 70–80% three weeks after the last mAb injection. Instead, it is possible that cytotoxic CD4^+^ T cells play a role in this vaccination model. This is also supported by the experiments performed in the, CD4^−/−^, CD8^−/−^ and CD4^−/−^CD8^−/−^ C57Bl/6 mice, discussed in more detail below.

Notably, CD4^+^ and CD8^+^ cell subsets could be depleted before immunization or tumour challenge in BALB/c mice, without any significant effect on inhibition of tumour growth, while the combined depletion in the induction phase abrogated tumour protection. The effect of the latter on the induction phase could however not be isolated from that on the effector phase, due to the prolonged depletion of these populations as mentioned above.

The reduced anti-tumour effect after injection of anti-asialo GM1 antibodies indicates that NK cells play a role. Asialo-GM1 is expressed on NK cells and on about 3% of CD8^+^ cells in BALB/c mice [21], while expression on CD4^+^ cells in this mouse strain has to our knowledge not been studied. Interestingly, after infection of mice with viruses such as respiratory syncytial virus and lymphocytic choriomeningitis virus, the expression of asialo-GM1 increases on both CD4^+^ and CD8^+^ cells. More than 90% of virus-specific CD4^+^ as well as CD8^+^ T cells can be asialo-GM1 positive [22], [23]. Whether upregulation of asialo-GM1 also occurs after inoculation with murine pneumotropic virus or MPtVLPs has not been examined, but it is possible that injection of anti-asialo GM1 antibodies in this study could also have had an effect on CD4^+^ and CD8^+^ cells. This hypothesis is possibly supported by the fact that the combined depletion effect on CD4^+^ and CD8^+^ cells was not enhanced by NK cell depletion.

Normal and CD4^−/−^, CD8^−/−^ single and double knock-out C57Bl/6 mice were also included in rejection studies. However, these systems were not as stringent since it was more difficult with C57Bl/6 EL4-Her2 both to obtain tumour outgrowth in control mice, and to achieve tumour protection in immunized mice. Depletion or knockout of both CD4^−/−^ and CD8^−/−^ cells abrogated protection against tumour outgrowth, in accordance to that discussed above in BALB/c mice. Similarly, CD8^−/−^ mice were protected. However, the immunization effect was lost in CD4^−/−^ mice, and since anti-Her2/*neu* antibodies were not shown here or previously [8], [11], [12], the results again suggest that CD4^+^ cytotoxic T cells could be active. When manipulating the system by depletion of CD4^+^ and/or CD8^+^ cells, it was easier to obtain an abrogation of the anti-tumour response compared to the BALB/c model. However, depletion of CD4^+^ cells, seemed again to result in a more profound effect.

Our present interpretation is that there is a redundancy of immune effectors after the very efficient Her2MPtVLP vaccination in BALB/c mice, and that when a single component is abolished a reasonable immune response is still obtained. In a more vulnerable system, i.e. in C57Bl/6 normal and knock-out mice abrogation of one cell type makes the mice sensitive to tumour outgrowth. Hence, Her2MPtVLP vaccination has the ability to activate several types of immune cells, and it is therefore necessary to deplete several immune components to abrogate the immune response completely.

To the very best of our knowledge, only one other study has in detail investigated the immune compartments *in vivo* responsible for tumor rejection following vaccination with cVLPs. Greenstone *et al* showed that cVLPs based on HPV16 induced protection against tumor outgrowth in normal and MHC class II-deficient mice as well as mice depleted of NK cells [10]. However, mice lacking β_2_-microglobulin or perforin were not protected implying that tumour protection was mediated by MHC class-I restricted cytotoxic T cells independent of CD4^+^ T cells [10]. Whether the same is true for cVLPs from murine polyomavirus and murine pneumotropic virus has never been studied. Although papillomaviruses and polyomaviruses are structurally similar, their VLPs differ. For instance, HPV VLPs induce maturation of dendritic cells [24] while most polyomavirus VLPs do not [11], [12], [24], [25] and they also bind to different cell surface receptors [26]–[29].

Other tumour vaccines have been studied more extensively in this context. Tumour protection after vaccination with vaccines based on DNA, dendritic cells or adenoviruses, is often lost following depletion of CD8^+^ cells during the effector phase [15], [30]–[37]. On the contrary, there are other studies with DNA vaccines, where elimination of CD4^+^ cells abolishes tumour protection in the effector phase [15], [31], [38], [39]. Depletion of NK cells has also been shown to abrogate protection in some DNA vaccination models [30], [38], [40]. However, dependence on NK cells is not as common as dependence on CD4^+^ and CD8^+^ cells.

Also with regard to studies on tumor vaccines in knock-out mice the results vary. In some studies with DNA vaccines in knock-out mice, the simultaneous presence of CD4^+^ and CD8^+^ cells is necessary [40]–[42]. However, one study with a vaccine based on vaccinia virus showed requirement for CD4^+^ cells [43], while another with an adenovirus-based vaccine showed dependence on CD8^+^ cells [44]. Another relevant study by Wen *et al* showed that immunity in mice against a methylcholanthrene induced fibrosarcoma was induced in both CD4^−/−^ and CD8^−/−^ single knock-out mice, but not in CD4^−/−^CD8^−/−^ double knock-out mice [45], which is then in line with our results in BALB/c mice. Hence, the results differ markedly between various studies and seem to depend both on the tumour antigen, the kind of vaccine and the animal model. It is therefore not obvious beforehand which immune compartments will mediate tumour rejection in a particular model, and every single model should be studied individually.

To investigate whether Her2MPtVLPs can induce long-term memory, immunization either with or without CpG was performed up to ten weeks before challenge. A statistically significant protection was obtained when Her2MPtVLPs combined with CpG were given as long as ten weeks prior to challenge. Although we have not confirmed that the T cells responsible for protection have a memory phenotype [46], [47], it is known that an immune response so long after immunization is normally mediated by memory T cells [46], [48], [49].

In conclusion, immunization with Her2MPtVLPs is efficient in stimulating several compartments of the immune system, and induces an efficient immune response including a memory T cell response. Hence, when depleting mice of isolated compartments, tumour protection is not as efficiently abolished as when depleting several immune compartments together.
